# The Anthrax Toxin Lethal Factor in Solution Does Not Have the Protein’s Crystallized Structure

**DOI:** 10.3390/toxins17040157

**Published:** 2025-03-22

**Authors:** Kenneth A. Rubinson, John J. Kasianowicz

**Affiliations:** 1 Department of Biochemistry and Molecular Biology, Wright State University, Dayton, OH 45435, USA; 2 NIST Center for Neutron Research, National Institute of Standards and Technology, Gaithersburg, MD 20899, USA; 3 Department of Physics, University of South Florida, Tampa, FL 33620, USA; 4 Freiburg Institute for Advanced Studies, Universität Freiburg, 79104 Freiburg, Germany

**Keywords:** anthrax, lethal factor, solution structure, SANS

## Abstract

The bacterium *Bacillus anthracis* secretes three protein exotoxins: Protective Antigen 83 (PA83), Lethal Factor (LF), and Edema Factor (EF). A cleaved form of PA83 (PA63) aids LF and EF entry into the cytoplasm, which leads to anthrax-induced cell death. The Protein Data Bank (PDB) has more than 25 structures of LF: the monomer alone, bound with inhibitors, or bound to PA63. The structures are all—with only minor shifts of a few Ångströms—nearly congruent. We have measured the structure of LF at equilibrium in D_2_O solution by small-angle neutron scattering (SANS). The shape is modeled well by a parallelepiped (all angles 90°) with dimensions of 12 Å × 49 Å × 129 Å. For a protein with a typical density of 1.4, the molecular weight would be between 55 and 94 kDa, which is comparable to that of the 90.2 kDa monomer. However, the LF crystal structure PDB 1pwu (a generally V-shaped molecule with equal arm lengths ≈ 70 Å) with the same model fits the dimensions 30 Å × 48 Å × 104 Å. Given the large changes in the long and short dimensions, straightforward physical modeling of the solution structure from the crystal form is unable to match the SANS results.

## 1. Introduction

Anthrax is a highly contagious disease caused by the Gram-positive zoonotic bacterium *Bacillus anthracis*. The highly virulent disease, which has been known for centuries [1], can enter humans by several different routes, including cutaneous, ingestion, and inhalation routes [2]. Even though the infection is potentially lethal at low doses (LD_50_ ≈ 8000 colony-forming units [3]), it persists in part because *B. anthracis* can remain dormant in soil for decades in a robust spore form [4]. Although naturally occurring anthrax-related infections have become less common, the bacillus has recently been used as a bioterrorism agent [5,6].

The molecular mechanism of anthrax infection is based on three protein exotoxins secreted by *B. anthracis*: Protective Antigen 83 (PA83), Lethal Factor (LF), and Edema Factor (EF) [7]. A cleaved form of PA83 (PA63) aids LF (≈90 kDa, 776 amino acids) and EF (≈89 kDa, 767 amino acids) entry into the cytoplasm, which leads to anthrax-induced cell death. The Protein Data Bank (PDB) lists more than 25 structures of LF: alone, bound to PA63, or as full or partial molecules bound to inhibitors and substrates. The structures are all-with only minor shifts of a few Ångströms-nearly congruent, as usefully and clearly shown in Antoni’s Figure 3 (doi.org/10.1371/journal.ppat.1008530.g003) [8].

A vigorous discussion has been ongoing for years about the mechanisms that lead to the transport of LF from the bloodstream into the cell cytoplasm, where the zinc endopeptidase LF inhibits downstream signal transduction pathways, resulting in cell death [9,10]. Most of these conversations make use of the LF crystal structure. The crystallized, unbound LF shows a generally V-shaped molecule with approximately equal arm lengths of about 70 Å (molecule 1 of PDB 1pwu). Also, while structures are generally sought to understand how molecules interact with other species, for anthrax toxins, it should be appreciated that they can also aid therapeutic agent development.

With the possibility of adding some clarity to this discussion by measuring the solution structure of LF, we obtained a modeled shape of LF at equilibrium in D_2_O solution (5 mmol/L HEPES, 50 mmol/L NaCl, and pD 7.5) by small-angle neutron scattering (SANS). The method makes use of the neutron scattering contrast between the protein and the D_2_O solvent [11]. The solution structure obtained with SANS vastly differs from all the solid-state structures. Details of the SANS experimental results and analysis are described below.

## 2. Results

### 2.1. Analysis of the SANS Scattering Curve

**Figure 1:**
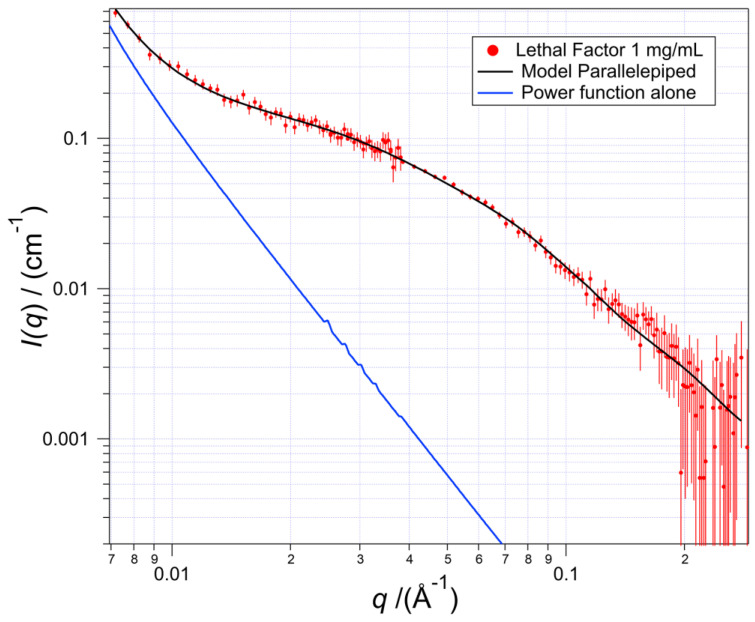
SANS scattering data with the calculated best-fitting scattering curve (solid, black) from the sum of scattering from a model homogeneous parallelepiped and a power-law function with the latter to fit scattering from a larger entity in the solution. The blue line shows the power-law function alone; that scattering as modeled comprises less than 10% of the total scatter at *q* = 0.02 and less than 1% at *q* > 0.04. The error bars show the standard deviations of the counting uncertainties.

The red data points in Figure 1 illustrate the experimental results in a log–log plot of the scattering intensity *I*(*q*) *versus* the momentum transfer *q*, which have units of cm^−1^ and Å^−1^, respectively. The best-fitting curve (*solid black*) was calculated from a sum of the scattering from a solution of a randomly oriented model homogeneous right parallelepiped in solution representing the structure of the Lethal Factor molecules plus a power-law function, *q*^-*n*^. The latter fits the scattering from some larger particle(s) because it occurs at the left side (longer lengths) of the *q* range. The power function alone is shown by the blue line. The value of the exponent *n* between 2.8 and 3.1 for the log–log slope fits the data range and characterizes scattering from a three-dimensional cluster. The explanation of the origins of values of the power-law slopes can be found in references [12,13,14].

**Figure 2:**
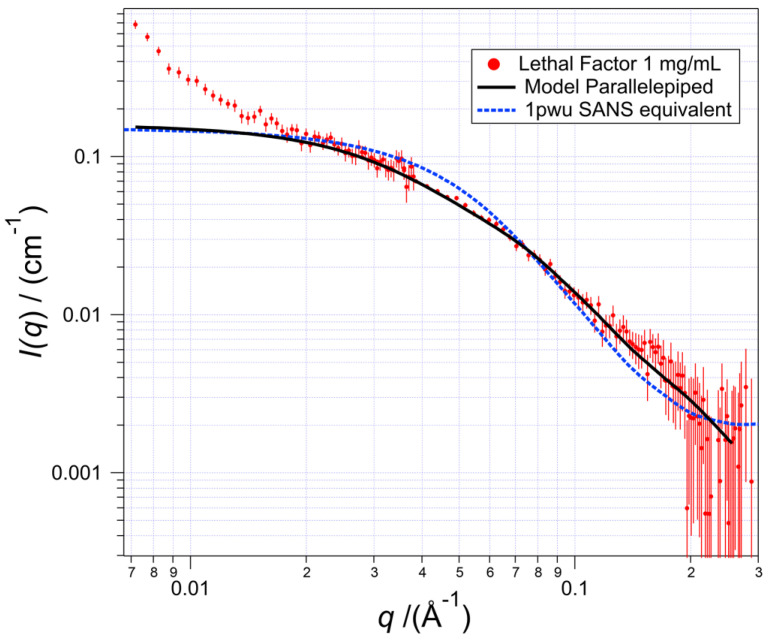
SANS scattering data with the calculated best-fitting scattering curve (solid, black) from the model parallelepiped-shaped scatterers that are homogeneous in their scattering length density and randomly oriented. The crystallized LF structure PDB 1pwu (molecule 1 of the unit cell) has its calculated SANS-equivalent scattering curve shown in dashed blue. This shows the significant difference in shape of the two structures.

The value of the power law that fits the low-*q* scattering influences the fit for the long edge of the parallelepiped model for LF because its intensity contributes about 10% of the scattering in that edge’s length range. When this power function is subtracted, the subsequent scattering curve is that of the non-aggregated LF structure alone, which is shown as the black line in Figure 2. Note that the contribution of the power-law function to the fit is <1% for *q* > 0.04 Å^−1^.

The parameters and uncertainties for the best-fitting parallelepiped as a geometric model for the Lethal Factor are shown in Table 1. The uncertainties listed are the limits found from numerous simulations that fit well over the entire data range. Given the noise level of the short distance (data at lower right), values of 0.0006 cm^−1^ to 0.0015 cm^−1^ could be used with little influence on the overall quality of fit but with the shortest side of the protein model strongly dependent on that background. These interplays can be seen in the values for the short edge reported in Table 1.

The Lethal Factor best fit gives a structure that is modeled by a parallelepiped with dimensions of 12 Å × 49 Å × 129 Å and the uncertainties listed in Table 1. This is a volume of 76 (+35, −11) × 10^3^ Å^3^. With a typical compact protein’s density of 1.4 [15], this volume is equivalent to a formula weight between 55 and 94 kDa.

### 2.2. The Molecular Properties Found by SANS Compared with Known LF

LF has a known molecular mass of 90.2 kDa [16], indicating from the calculated molecular mass that the scatterers are unassociated LF molecules. The larger volume of the 1pwu crystal structure shown in Table 1 indicates that the fitting parallelepiped has a volume that is not well filled by the protein, which at that density would have a molecular mass of 124 kDa, i.e., 37% greater than the accepted value. It is interesting that the gene for LF codes for a protein with 809 residues and a molecular mass of 93,969 Da. This includes 33 residues that form a ribosome binding site—a signal peptide—that is cleaved to form the 90.23 kDa active protein [16].

## 3. Discussion

### 3.1. The Crystal Structure and Solution Structure Compared

Note that the neutrons scatter from nuclei, and the calculated dimensions are for the distance from nucleus to nucleus. To compare them with X-ray and molecular measurements, an approximate 1 Å radius should be added to each atom, so the distances for comparison should be 2 Å longer than those listed in Table 1. Such adjustments are significant only for the shortest distance.

Comparing the two sets of dimensions shown in Table 1, especially because of the differences in the shortest one, it is conceivable that a local hinge has been opened, but there is no such obvious region visible in the crystal structure. As a result, straightforward physical modeling of the solution structure from the crystal form is unable to match the SANS result. We are unaware of any sets of programs/algorithms that can suggest a structure with this measured, quantitative final shape that differs so greatly from a crystal structure and where there is such a wide range of possibilities given only the primary sequence. However, there may be some promise with modifications of AlphaFold, namely, AFsample 2 [17] and AF-Cluster [18], that probe a wider range of potential structures for a given a primary sequence.

### 3.2. Perspective on the Lethal Factor’s Properties

An extensive literature exists about the structure and properties of the anthrax Lethal Factor. But because the structure we have discovered here is that of the full-length LF, we only include and compare prior reports relating to the entire molecule and not to some fractional part of the protein. 

The fact that we find the flattened, elongated structure in solution contradicts beliefs that the “... apo-LF already exists as a compact structure, and its binding to PA does not alter its domain arrangement” [19]. Given that the present study provides the only solution structure of LF measured under near-physiological conditions, it is not clear what conditions cause the structures seen in the solid state to form. While there have been statements made that some modest flexibility in LF exists [20], the structure here and measurements made in vitro by Baker et al. [21] show that the structure can vary significantly.

The Baker group [21] measured LF transported through hydrogel membranes with differing average pore sizes under a range of transmembrane voltages, with the pore characteristics simultaneously tested with other proteins of various masses. The germane lesson is that the 90.2 kDa LF more easily passed electrophoretically into ≈2.4 nm diameter pores than numerous lower-molecular-weight globular proteins, namely, green fluorescent protein (27 kDa), Annexin V (36 kDa), ovalbumin (45 kDa), and bovine serum albumin (67 kDa). With direct comparisons between the LF and the other proteins, the possible heterogeneity of pore sizes as well as the possibility that a few larger pores might be responsible are greatly minimized if not eliminated. These experiments also showed that proteins retaining some secondary structure such as α-helices pass more easily than fully denatured entities. This important set of experiments further indicates the relatively low stability of the higher-level structures of LF relative to common globular proteins.

These facts should call into question the common basis used for discussion of whether LF transports through the pore of the anthrax PA63 [22] (but see [23,24]) and, if so, how that ambiguity of structure might confuse the chemistry leading to the etiology of the cellular damage involved in the disease.

## 4. Conclusions

Essentially all discussions of the mechanism of bringing the anthrax Lethal Factor from the bloodstream into the cellular interior to cause cell death in a manner representative of this toxin have assumed that the crystal structure exemplifies the stable form in solution. SANS scattering of full-length Lethal Factor shows an equilibrium structure significantly longer and flatter, but with the same width as that of the crystal structures. Importantly, the *B. anthracis* Lethal Factor has been shown to pass into gel membrane pores electrophoretically far more easily than globular proteins of smaller size, including those with less than one-third the mass [21].

These results also set a challenge for biomolecular calculations to find stable structures that are significantly different from any known structure but with tight constraints on the shape of the resultant configuration. These calculations are difficult given the huge number of possibilities while also in the end adhering to the well-known rules of protein packing and secondary structure formation. Such a calculation appears to be a difficult and new type of task.

## 5. Materials and Methods

### 5.1. Lethal Factor Solution

Lyophilized, recombinant anthrax Lethal Factor (LF; Cat. 172B, List Biological Laboratories). To the material was added 99.9% D_2_O (Cambridge Isotope Labs) to make a solution of 1.0 mg mL^−1^ in the protein. The final solution additionally had 5 mmol/L HEPES, 50 mmol/L NaCl, and pD 7.5. This was produced by adding 1.0 mL D_2_O to the vial contents as purchased.

The active LF molecule has 776 residues with a molecular mass of 90.23 kDa. The List Biological Laboratories’ recombinant LF used here has two additional residues (histidine and methionine) on the N-terminal, giving it a molecular mass of 90.49 kDa. We assumed that this addition has a negligible influence on the overall structure.

### 5.2. Small-Angle Neutron Scattering (SANS) Data Collection

The 1 mg mL^−1^ solution was held in a (1.00 ± 0.01) mm pathlength cylindrical silica spectrometry cell (Hellma, Plainview, NY, USA) with a volume of 320 μL. SANS measurements were performed on the NG7 30 m SANS instrument at the NIST Center for Neutron Research (NCNR) in Gaithersburg, MD [25]. Data were collected for both the LF solution and the D_2_O solvent at ambient temperature. For the data collection, the neutrons were selected with wavelength λ = 6.0 Å, and Δλ/λ = 0.11.

Scattered neutrons were detected with a 64 cm × 64 cm two-dimensional position sensitive detector with (128 × 128) pixels and 0.5 cm resolution per pixel. Data reduction was accomplished using Igor Pro software version 7 (WaveMetrics, Lake Oswego, OR, USA) with SANS macros developed at the NCNR [26]. Raw counts were put on the same relative scale by normalizing to an incident beam monitor count made by a detector in parallel with the data collection. The scattering was then corrected for non-uniform detector-pixel response. Data were placed on an absolute scale by normalizing the scattering intensity to the measured incident beam flux. Finally, the data were radially averaged to produce the scattering curves of *I*(*q*) versus *q*—where *q* = (4π sin θ/λ), λ is the de Broglie wavelength of the neutron and 2θ is the scattering angle measured from the axis of the incoming neutron beam. Two camera positions were used: 2 m and 10 m. A 25 cm detector offset for the 2 m position, together with the 6.0 Å wavelength, provided a *q* range of 0.006 Å < *q* < 0.3 Å^−1^, equivalent to a length range of ≈1000 Å to ≈21 Å.

The LF scattering data were corrected for the D_2_O background by subtracting the scattering from D_2_O solvent. No solute-volume correction was needed for the 1 mg mL^−1^ solution since any adjustment would have been less than 0.1%, which is within the uncertainties of the scattering amplitudes.

The neutron optics was accounted for by a smearing algorithm that was applied in the data reduction.

### 5.3. Scattering Curve Fitting

Neutron scattering occurs when there is a contrast between the solvent and the solute; this is the equivalent to a dielectric constant difference for electromagnetic radiation. The variable expressing the magnitude of the scattering is called the scattering length density, abbreviated SLD, which has units of cm^−2^. A right parallelepiped (all angles 90°) with a homogeneous SLD was used as a simple geometric model for the LF molecular shape in the buffer. A parallelepiped was used to keep the three spatial lengths orthogonal in the data fitting protocol, unlike, e.g., an ellipsoid. The *q* range fitted is shown in Figure 1 and omits scattering from aggregated structures that scatter the low-*q* range of the data, *q* ≲ 0.02 Å^−1^, equivalent to lengths longer than ≈ 300 Å.

### 5.4. Calculating the SANS Scattering Equivalent to the Crystal Structure

The SANS scattering curve expected from solitary LF molecules was calculated using molecule 1 of the two molecules in the PDB structure 1pwu by using the program XTAL2SAS, which is in the section ‘retired’ of the Sassie (http://sassie-web.ibbr.umd.edu/sassie2/) set of programs for small-angle scattering. In the program, a homogeneous sphere representing each amino acid is positioned according to the α-carbon coordinates. The sphere that represents the amino acid is assigned a size and SLD calculated from the known amino acid volume and its chemical composition at any H_2_O/D_2_O ratio using the values of Jacrot [27]. This representative structure is placed in a mathematical box, with the option to include a region of bound water up to 5 Ångströms thick, again with a selected H_2_O/D_2_O ratio. Points are then generated at random within the box. If a point is contained within the model structure, it is saved. Distances between every pair of saved points are weighted according to the product of the SLD at each point [25,28]. The distance distribution function, *P*(*r*), is found by summing all possible distances between pairs of points. Then, the estimated *I*(*q*) is calculated from *P*(*r*) by performing a Fourier transform. The 1pwu-equivalent *I*(*q*) curve was then scaled, and a fixed background added to bring it into a match-by-eye to the SANS data. A quantitative best fit is not useful because the difference with the experimental curve is so great, such that numerous possible decent fits are possible. For the fits shown in Figure 2, the χ^2^ for the homogeneous parallelepiped and the 1pwu x-ray structure’s calculated scattering were 0.211 and 0.535, respectively.

The crystal structure does not resolve the first 29 amino acids of the LF polypeptide chain. This absence was ignored with the judgement that this 3.7% of the total chain will not distort the calculated SANS curve used to illustrate the large structural difference.

## Figures and Tables

**Table 1:** Dimensions of the best-fitting homogeneous right parallelepiped ^a^.

Parallelepiped Model	Shortest/Å	Middle/Å	Longest/Å	Volume/Å^3^	MW for ρ = 1.4	(*R*_g_/Å) ^b^
SANS data	12(+4.5,−1)	49 ± 1	129 ± 6	76(+35,−11) × 10^3^	65(+29,−10) kDa	42 ± 2
1pwu	≈30	≈48	≈104	≈150 × 10^3^	124 kDa	34

^a^. Uncertainties include the full range of values found from numerous optimal fittings showing a range of power-law exponent values (2.8 to 3.1) that fit the data and a range of background intensities (0.0006 to 0.0015) that, because of their small values, have little effect on the overall quality of fit. ^b^. *R*_g_ values were found with a calculator at https://scattering.tripod.com/xitami/java/rgcalc.html (accessed on 18 March 2025).

## Data Availability

Data can be accessed at https://dx.doi.org/10.18434/T4201B?urlappend=ng7sans/200806/NG7SANS14/data/ (accessed on 18 March 2025). Reduced and corrected SANS scattering data supporting the findings of this study are available from the corresponding author upon reasonable request.
